# Health Gains and Financial Protection Provided by the Ethiopian Mental Health Strategy: an Extended Cost-Effectiveness Analysis

**DOI:** 10.1093/heapol/czw134

**Published:** 2016-10-09

**Authors:** Kjell Arne Johansson, Kirsten Bjerkreim Strand, Abebaw Fekadu, Dan Chisholm

**Affiliations:** 1 Department of Global Public Health and Primary Care, University of Bergen, Norway; 2 Department of Addiction Medicine, Haukeland University Hospital, Bergen, Norway; 3 College of Health Sciences, School of Medicine, Department of Psychiatry, Addis Ababa University, Addis Ababa, Ethiopia; 4 Department of Psychological Medicine, King’s College London, Institute of Psychiatry, London, UK; 5 Department of Mental Health and Substance Abuse, World Health Organization, Geneva

**Keywords:** Equity, ethics, mental health, poverty reduction strategy papers, priority setting

## Abstract

**Background:** Mental and neurological (MN) health care has long been neglected in low-income settings. This paper estimates health and non-health impacts of fully publicly financed care for selected key interventions in the National Mental Health Strategy in Ethiopia for depression, bipolar disorder, schizophrenia and epilepsy.

**Methods:** A methodology of extended cost-effectiveness analysis (ECEA) is applied to MN health care in Ethiopia. The impact of providing a package of selected MN interventions free of charge in Ethiopia is estimated for: epilepsy (75% coverage, phenobarbital), depression (30% coverage, fluoxetine, cognitive therapy and proactive case management), bipolar affective disorder (50% coverage, valproate and psychosocial therapy) and schizophrenia (75% coverage, haloperidol plus psychosocial treatment). Multiple outcomes are estimated and disaggregated across wealth quintiles: (1) healthy-life-years (HALYs) gained; (2) household out-of-pocket (OOP) expenditures averted; (3) expected financial risk protection (FRP); and (4) productivity impact.

**Results:** The MN package is expected to cost US$177 million and gain 155,000 HALYs (epilepsy US$37m and 64,500 HALYs; depression US$65m and 61,300 HALYs; bipolar disorder US$44m and 20,300 HALYs; and schizophrenia US$31m and 8,900 HALYs) annually. The health benefits would be concentrated among the poorest groups for all interventions. Universal public finance averts little household OOP expenditures and provides minimal FRP because of the low current utilization of these MN services in Ethiopia. In addition, economic benefits of US$ 51 million annually are expected from depression treatment in Ethiopia as a result of productivity gains, equivalent to 78% of the investment cost.

**Conclusions:** The total MN package in Ethiopia is estimated to cost equivalent to US$1.8 per capita and yields large progressive health benefits. The expected productivity gain is substantially higher than the expected FRP. The ECEA approach seems to fit well with the current policy challenges and captures important equity concerns of scaling up MN programmes.

Key Messages

- The National Mental Health Strategy in Ethiopia for depression, bipolar disorder, schizophrenia and epilepsy is estimated to cost equivalent to US$1.8 per capita and to yield large progressive health benefits.
- A 78% overall rate of return (US$50M annually) to investment is expected from depression treatment in Ethiopia due to productivity gains.
- Universal public finance provides minimal financial risk protection because of the low current utilization of mental and neurological services in Ethiopia.

## Introduction

High quality health service delivery for mental and neurological (MN) disorders in low-income settings is likely to bring large health and non-health outcomes. Treatment demand is high and current coverage is low. Depression, schizophrenia, bipolar disorders and epilepsy cause around 13% of all Years of Life Lost due to Disability (YLD) in Sub-Saharan Africa (SSA) according to 2013 estimates (Global Burden of Disease Study, 2015). Little is known about the return on investing in MN programmes in low-income countries. Such information is needed for making evidence based investments in MN health care. We aim to explore a novel approach for measuring equity relevant policy impacts of scaling up MN services in one particular low-income country.

Ethiopia is used as a case for testing how health and non-health outcomes could be measured. There are only 0.4 specialists in psychiatry per one million population in Ethiopia (Strand *et al*. 2015). The annual total health budget in Ethiopia is low (US$25 per capita) (World Bank and World Development Indicators). The National Mental Health Strategy in Ethiopia specifies a massive scale-up of psychiatric and psychological care during the next decade (Saxena *et al*. 2007; mhGAP-Ethiopia Working Group, 2010; Bruckner *et al*. 2011; Federal Democratic Republic of Ethiopia Ministry of Health, 2012). Shortage of human resources, low health budgets and ambitious policy goals stresses the need for evidence on the opportunity cost of MN interventions in Ethiopia, as well as other low-income settings (Jamison *et al*. 2013). The importance of both efficient and equitable scale-up of mental health care is explicitly recognized in the suggested scale-up of services for MN disorders.

Standard cost-effectiveness analyses (CEAs) are relevant for making rank orders on which interventions that maximizes health outcomes the most (Drummond *et al*. 2005). However, equity concerns are not explicitly addressed in CEAs (Norheim *et al*. 2014; Brock and Wikler, 2006; Jamison *et al*. 2013). Information on health inequality among income groups and medical impoverishment are important in addition to cost-effectiveness (Brock 2003; Daniels 2008). Direct out-of-pocket (OOP) payments affect those least able to afford care and are an important risk factor for health-care-induced impoverishment. The reduction or elimination of private OOP expenditures to health care can represent major financial savings for affected households. Public financing of health service costs can also increase the use of services, especially for those whose incomes are so low that they do not access services in the first place. Prepayment mechanisms, such as national or social insurance, create safety nets for at-risk populations from the adverse financial consequences of mental disorders. Information on efficient purchase of equity concerns like financial risk protection (FRP) and distribution of benefits across income groups is needed in evidence-based policy decision making (World Health Organisation, 2014).

Our application of extended cost-effectiveness analysis (ECEA) to MN disorders focuses on universal public financing as an instrument for FRP (Verguet *et al*. 2015a, b). Public financing provides FRP benefits to households by reducing the financial burden due to disease and the impoverishment-related consequences of the covered health care service. A large proportion of total health spending in Ethiopia is currently from OOP expenditures, the estimates vary between 30-40% over the last ten years (World Bank and World Development Indicators, Federal Democratic Republic of Ethiopia Ministry of Health, 2014). ECEA take the distribution of household costs and health outcomes across different socioeconomic groups in the population into account, but also explicitly examines the extent to which interventions or policies protect households against the financial risk of medical impoverishment (Verguet *et al*. 2015a,b). Important equity concerns can be integrated into policy decision-making quantitatively by ECEA methods. Few ECEAs are available for mental health care.

The basic scale-up scenario in the National Mental Health Strategy in Ethiopia targets treatment for depression, psychosis, bipolar disorder and epilepsy; key interventions in the World Health Organization (WHO) mental health Gap Action Programme (mh-GAP) (mhGAP-Ethiopia Working Group, 2010). Recent evidence on cost-effectiveness of the basic scale-up scenario indicates that treatment of depression, bipolar disorder, epilepsy and schizophrenia cost between US$300 and US$2000 per Disability Adjusted Life Year (DALY) averted (Reinap *et al*. 2005; World Health Organization, 2006; Gureje *et al*. 2007; Ayuso-Mateos *et al*. 2008; Chisholm *et al*. 2008; Salomon *et al*. 2012; Chisholm and Saxena 2012; Strand *et al*. 2015). Antipsychotics for schizophrenia are in the upper cost-effectiveness range and phenobarbital for epilepsy is in the lower cost-effectiveness range.

The objective of this paper is to apply ECEA methods to evaluate scale-up and universal public finance – government financing of all intervention costs irrespective of who is receiving care – of an MN package of interventions that are specified as a key in the National Mental Health Strategy in Ethiopia. With universal public finance, households would receive treatment of epilepsy, depression, schizophrenia and bipolar disorders free of charge at the point of care. Since this approach of extending results from an existing CEA is new and there are a few applications to MN disorders, we intended to test the applicability of this method.

## Methods

We use ECEA methods (Verguet *et al*. 2013, 2015a,b) to evaluate the health and non-health impacts of increased coverage of the MN treatment package: phenobarbital for epilepsy, fluoxetine combined with cognitive therapy and proactive case management for depression, valproate combined with psychosocial therapy for bipolar affective disorder, and first-line antipsychotic medication (haloperidol or chlorpromazine) plus psychosocial treatment for schizophrenia. Interventions in the analysed packages were selected in accordance with recommendations in the National Mental Health strategy in Ethiopia (Federal Democratic Republic of Ethiopia Ministry of Health, 2012). All selected interventions have been analysed in an existing standard CEA contextualized for an Ethiopian setting, and in this ECEA we analyse the interventions that were found to be most cost-effective for each condition (Strand *et al*. 2015). The disease-specific incremental cost-effectiveness ratio (ICER) for each of the selected interventions is estimated by (Strand *et al*. 2015) to be: US$321 (phenobarbital for epilepsy); US$1026 (fluoxetine combined with cognitive therapy and proactive case management for depression); US$2023 (valproate combined with psychosocial therapy for bipolar affective disorder); and US$2001 (first-line antipsychotic medication plus psychosocial treatment for schizophrenia).

The ECEA builds on the parent CEA of MN health care in Ethiopia (Strand *et al*. 2015). The existing model is a generalized WHO-CHOICE CEA (World Health Organisation and WHO-CHOICE, 2016) that is contextualized to an Ethiopian setting. A mix of primary cost data and secondary data sources were used in the original CEA (regional WHO-CHOICE dataset and empirical literature). More details on this population-based multi-state analytical health economic model can be found in the CEA study (Strand *et al*. 2015).

### Healthy life years across income groups

In this ECEA, health benefits are measured in healthy life years gained from interventions as compared to a null scenario if no interventions are scaled-up. Treatment effects are incremental reductions in case fatality, prevalence or disability weight, or increased remission rates, by the respective MN interventions. We split the Ethiopian population into five income quintiles and run the existing analytical model (Strand *et al*. 2015) for each income group with quintile-specific prevalence rates. Table 1 shows details on parameter assumptions. The model has a life table structure that includes disability weights to estimate healthy life years (World Health Organisation and Department of Health Statistics and Information Systems, 2014). The interventions are implemented over a 10-year period, but health benefits are counted over a lifetime. Healthy life years are discounted at 3% and no age-weights are used.

**Table 1:** Parameters used for the extended economic evaluation of universal public finance (UPF) for the National Mental Health Strategy in Ethiopia

Parameter	Value	Reference
**Epidemiology/Demography**		
Prevalence mental disorders across wealth strata (poor; average; rich)	0.220; 0.135; 0.114	(Fekadu *et al*. 2014)
Treatment demand (prevalence)		(Global Burden of Disease Study 2015)
− Depression (age 15–29; 30–44; 45–60)	0.062; 0.068; 0.070	
− Bipolar disorder (age 15–29; 30–44; 45–60)	0.009; 0.012; 0.024	
− Schizophrenia (age 15–29; 30–44; 45–60)	0.002; 0.006; 0.006	
− Epilepsy (age 15–29; 30–44; 45–60)	0.007; 0.006; 0.006	
Population size (in millions, age 15–29; 30–44; 45–60)	29.1m; 15.8m; 8.1m	(UN Population Division 2015)
**Interventions**		
Efficacy:		(Strand *et al*. 2015)
− Depression (SSRI, CBT, proactive case management)	−31% disability/-38%remission/-35%incidence	
− Bipolar disorder (valproate and psychosocial therapy)	−65% disability/-65% case fatality	
− Schizophrenia (haloperidol plus psychosocial treatment)	−23% disability	
− Epilepsy (phenobarbital)	−43% disability/-60% remission	
Target coverage of interventions:		(Federal Democratic Republic of Ethiopia Ministry of Health 2012)
− Depression (by quintile, Q1-Q5)	0.3;0.3;0.3;0.3;0.3	
− Bipolar disorder (by quintile, Q1-Q5)	0.5;0.5;0.5;0.5;0.5	
− Schizophrenia and epilepsy (by quintile, Q1-Q5)	0.75;0.75;0.75;0.75;0.75	
− Epilepsy (by quintile, Q1-Q5)	0.75;0.75;0.75;0.75;0.75	
**Costs**		
Hospitalization cost per patient admitted (2010 US$)		
− Depression (utilization at this level)	US$538 (0.03)	(Strand *et al*. 2015)
− Bipolar disorder (utilization at this level)	US$330 (0.08)	
− Schizophrenia (utilization at this level)	US$1,777 (0.47)	
− Epilepsy (utilization at this level)	US$275 (0.11)	
Outpatient clinic cost per visit (2010 US$)		
− Depression (utilization at this level)	US$101 (0.25)	(Strand *et al*. 2015)
− Bipolar disorder (utilization at this level)	US$74 (0.31)	
− Schizophrenia (utilization at this level)	US$95 (0.50)	
− Epilepsy (utilization at this level)	US$85 (1.00)	
Primary care (health center/health post), cost per visit (2010 US$)		
− Depression (utilization at this level)	US$133 (1.00)	(Strand *et al*. 2015)
− Bipolar disorder (utilization at this level)	US$64 (0.50)	
− Schizophrenia (utilization at this level)	US$123 (0.50)	
− Epilepsy (utilization at this level)	US$46 (1.00)	
Gini index	0.3	(World Bank and World Development Indicators, 2015)
GDP (2014 US$, million)	US$54,798	
GDP per capita (2014 US$)	US$565	
Total societal income per capita (US$, by quintile Q1–Q5)	US$180; US$340; US$500; US$690; US$1110	
Total societal income per capita aged 15-60 (US$, by quintile Q1–Q5)	US$330; US$630; US$910; US$1260; US$2040	
Utility function as a function of individual income y	$\frac{y^{1-r}}{1-r}$ with r=3	(Verguet *et al*. 2015a, McClellan and Skinner 2006)

There is one model for depression, one for bipolar disorder, one for schizophrenia and one for epilepsy. The population in each of these models is divided into three health states (disease X, susceptible without disease X and dead). Transitions between health states occur annually and are determined by the disease-specific prevalence, remission rates, case fatality rates and age-specific mortality rates. The average age-specific disease prevalence used in the standard CEA (Strand *et al*. 2015) is adjusted to income-quintile-specific prevalence rates, using a population-based prevalence study conducted in Ethiopia (*n* = 1,497) (Fekadu *et al*. 2014). For each disorder, based on data extracted from (Fekadu *et al*. 2014), we obtain a prevalence ratio by income quintile (poorest-quintile, 1.4; second-poorest, 1.2; middle-quintile, 1; second-richest quintile, 0.8; and richest-quintile, 0.6) and apply this to the mean age-specific prevalence of each disorder. Disease-specific mortality, disability weights, intervention coverage and intervention effectiveness are held constant in each income group.

Current treatment coverage for all disorders is < 5% (Strand *et al*. 2015). Following the introduction of universal public finance, and in line with the National Mental Health Strategy, coverage for all income groups is modelled to reach 75% for treatment of schizophrenia and epilepsy, 50% for treatment of bipolar disorder and 30% for treatment of depression (Federal Democratic Republic of Ethiopia Ministry of Health 2012). Target coverage for depression is lower than the target coverage for the other interventions because the relatively high prevalence and low detectability of depression. Estimates of the efficacy of interventions were drawn from systematic reviews, meta-analyses and randomized controlled (see Table 1) trials (full details can be found in (Strand *et al*. 2015)).

### Health provider costs

Unit costs (US$2010) from the original CEA are used (see Table 1) and converted to US$2014 by a consumer price index GDP deflator (World Bank and World Development Indicators 2015). The original CEA has a health provider perspective on costs. By large, unit prices (e.g. lab costs, pharmaceuticals, salaries) and quantities needed at the various delivery platforms draw on data from the Amanuel Psychiatric Hospital (the only psychiatric hospital in Ethiopia at the time data were collected) and the International Drug Price Indicator Guide (http://erc.msh.org). Costs for planning and administration, training of staff and monitoring and evaluation at a national, provincial and district level are included in the total cost. Total costs are counted over the 10-year period that interventions are implemented and are discounted at 3%.

### Household financial burden

Depression, schizophrenia, epilepsy and bipolar disorder impose a financial burden on households. First, we quantify what households would pay due to illness-related cost in the absence of the programme (as it is today). Since the current coverage of mental health care is low in Ethiopia, the mental health programme is expected to represent very little cost savings from a household perspective. Before the MN programme is introduced, we assume that individuals with access to MN care pay OOP for 34% of all provider costs for treatment that currently is available (the national average OOP expenditures on health services in Ethiopia) (World Bank and World Development Indicators, Federal Democratic Republic of Ethiopia Ministry of Health, 2014). The government finances the remaining 66% of MN health care costs. The treatment demand varies by income group in accordance to the prevalence distribution. Age- specific prevalence was updated according to recent GBD2013 estimates (Global Burden of Disease Study, 2015). Second, we estimate the private expenditures averted by the universal public finance of MN treatments and reducing the existing OOP expenditures to 0% for each income quintile.

### Financial risk protection

The approach applied for estimating FRP is described in great detail elsewhere (Verguet *et al*. 2013 , 2015a). A standard utility-based model is applied to quantify what may be seen as a ‘fair’ societal risk premium, where universal public financing of MN care is considered as a social insurance programme. We calculate the insurance value of universal public finance of the Ethiopian MN policy by using a money-metric-value of insurance as the outcome unit of FRP (Verguet *et al*. 2015a; McClellan and Skinner 2006). This US$value represents how much the society is willing to pay for eliminating the financial risk individuals currently face due to MN disease. Universal public finance delivers FRP benefits to patients by averting the existing OOP expenditures associated MN disorders. First, we estimate the expected individual income before universal public finance of MN services by a function based on (McClellan and Skinner 2006; Verguet *et al*. 2015a,b):

$$E_{J}(y)=p_{J}Cov\left(y-c_{J}\right)+\left(1-p_{J}Cov\right)y$$

where *p* is the probability of getting a MN disease, *Cov* is the current treatment coverage, *c* is the OOP expenditures to MN treatment, *y* is income in quintile *J*. See Table 1 for details on the parameters that are used as input. Second, we estimate the certainty equivalent for the same individual, *Y*_j_*, by:

$$Y_{J}^{*}=\;U^{-1}[p_{J}U\left(y-c_{J}\right)+\left(1-p_{J}\right)U(y)]$$

where *U* is a constant relative risk aversion utility function (Table 1). The certainty equivalent estimates the amount of money the individual is willing to have in order to obtain certainty in the expected OOP expenditures averted from universal public finance. Third, total money-metric-value of insurance in the quintile *J* is then calculated by:

$${\text{In}s}_{J}=\int_{J}^{}(E_{J}(y)-Y_{J}^{*})f\left(y\right)dy.$$

where f(y) is the income distribution in the population proxied by a Gamma density based on the GDP per capita and Gini index in Ethiopia (Table 1) (Salem and Mount 1974). The insurance value is simply the difference between the expected value of income before universal public finance of MN services and the certainty equivalent.

### Productivity gains

Treatment of MN disorders is likely to provide other important welfare gains, in particular productivity at the household and societal levels. Therefore, and because we expected low FRP due to the low current level of utilization of MN services, we explore the expected productivity gains from scaling up the provision of depression care and treatment to productive ages (age 15-60). We concentrate on depression in this age group because the disease burden is high in Ethiopia, and evidence indicates that depression has a substantial impact on productivity (Clark *et al*. 2009, Goetzel *et al*. 2003). Around 6% of the adult Ethiopian population is estimated to have a depressive episode at any given time (Table 1), with an average duration of 8.4 months (Strand *et al*. 2015). Productivity is lost during such episodes because of increased absence from work (absenteeism) and decreased work performance when present at work (presenteeism) (Goetzel *et al*. 2003). Depression treatment programmes have been shown to improve rates of employment by up to 5% in the United Kingdom (Clark *et al*. 2009). In the United States, costs associated with presenteeism have been estimated to be higher than the costs of treatment (Goetzel *et al*. 2003).

To estimate the productivity impact across income groups from scaling up treatment of depression in Ethiopia, we first adapt the Goetzel *et al*. (2004) approach to presenteeism to the context of Ethiopia. We use epidemiological, demographic, efficacy and cost data from the contextualized CEA of mental health care in Ethiopia (Strand *et al*. 2015) and updated data if available (see Table 1). The average reduction in duration of a depressive episode due to treatment was estimated to be 2.9 months (8.4 months * efficacy of 0.35). Second, this reduction in duration was converted to a reduction in absenteeism. Disability days (per month) due to depression are estimated to be 2.9 in low-income settings (Alonso *et al*. 2011). Hence, we assumed treatment would reduce the number of disability days by 8.7 days in total (2.9*2.9) in Ethiopia. Subsequently, population with depression, target coverage (30%) and an average daily income (per wealth quintile in the productive age groups (age 15–60) were multiplied by this change in absenteeism (8.7 days) to derive an estimate of the potential productivity gains in Ethiopia. In addition, we made an adjustment that took into account that losses in presenteeism were reduced by treatment. Patients with depression were found to have 3.7 days with partial disability per month in low-income countries (Bruffaerts *et al*. 2012). Partial disability means that on-the-job productivity is reduced because of disease. It was assumed that patients with depression had 1.2 full days lost per month because of presenteeism, based on the assumption that each partial day is equivalent to one-third of a full lost day. Subsequently, the associated productivity gain was estimated using the same method as for absenteeism.

All analyses were conducted using the R statistical package (www.r-project-org) and PopMod developed by WHO-CHOICE. Ethical clearance was obtained from the Institutional Review Board at the Medical Faculty of Addis Ababa University.

## Results

The expected annual cost of implementing the defined MN health care package at specified target coverage levels is approximately US$177 million (Table 2) for the whole country, equivalent to around US$1.8 per capita. The return on this investment in total population health gain exceeds 155 000 healthy life-years (Table 2), the majority of which derives from treatment of depression and epilepsy. The eliminated out-of-pocket spending by universal public financing is low in Ethiopia (around US$1 million in total) due to the low current utilization of MN health services (< 5%). The return in FRP is also extremely low, US$1,720 in total, for the same reasons. However, the expected productivity gain of depression treatment is substantially higher compared to the expected FRP. Scaled-up depression treatment at 30% coverage is expected to return total productivity gains of around US$50.7 million per year in Ethiopia (Table 3), which is close to 78% of the expected total cost of the depression treatment programme.

**Table 2:** Dashboard of the annual expected outcomes from scaling up the mental and neurological health care package in Ethiopia

Outcome	Income quintile					**Total**
	I	II	III	IV	V	
*Total cost of care (2014 US$, in 1 000, at target coverage)*^a^^,^^b^						
Schizophrenia	8 329	7 250	6 171	5 091	4 011	30 852
Bipolar disorder	11 988	10 435	8 881	7 327	5 772	44 404
Depression	17 467	15 247	13 013	10 766	8 506	65 000
Epilepsy	10 143	8 832	7 666	6 205	4 082	36 928
*Healthy life-years gained (at target coverage)*^b^						
Schizophrenia	2 420	2 100	1 790	1 480	1 160	8 956
Bipolar disorder	5 480	4 770	4 060	3 350	2 640	20 306
Depression	16 390	14 350	12 290	10 210	8 090	61 332
Epilepsy	17 680	15 420	13 260	10 860	7 270	64 502
*Private expenditures averted (2014 US$, in 1 000, at current coverage)*^c^						
Schizophrenia	22	19	16	13	11	81
Bipolar disorder	65	57	48	40	31	241
Depression	44	38	32	27	21	162
Epilepsy	149	130	113	91	60	544
*Insurance value (2014 US$, at current coverage)*^d^						
Schizophrenia	0.9	0.3	0.2	0.2	0.1	1.6
Bipolar disorder	38	13	7	7	3	67
Depression	113	40	22	21	9	206
Epilepsy	835	271	154	141	42	1 443

a Total costs = (direct government expenditures) + (private expenditures, including out-of-pocket costs).

b Target coverage associated with enhanced public financing for all income groups was set at 30% for depression treatment, 50% for bipolar disorder and 75% for the other two disorders.

c Private expenditures averted = out-of-pocket spending that is eliminated by switching to public financing.

d Insurance value = financial risk protection provided, based on current coverage.

**Table 3:** Expected productivity impact and net societal cost (2014 US$) of scaled-up depression treatment to 30% coverage

Cost/outcome	Income quintile					Total population
	I	II	III	IV	V	
Government cost of depression treatment programme (US$, million)	−17.5	−15.2	−13.0	−10.8	−8.5	−65.0
Productivity gain from scaled-up depression treatment (US$, million)^a^						
due to absenteeism	3.8	6.3	7.5	8.3	10.0	35.9
due to presenteeism	1.6	2.6	3.1	3.4	4.1	14.8
Net societal cost of depression treatment programme (US$, million)^b^	−12.0	−6.3	−2.4	−0.9	5.6	−14.3

a Total societal income per capita in productive ages (15-60) (2014) in Ethiopia is US$1,034: by quintile, US$330 for QI, US$630 for QII, US$910 for QIII, US$1260 for QIV and US$2,040 for QV.

b Net societal cost = (governmental cost) − (productivity gain).

**Figure 1:**
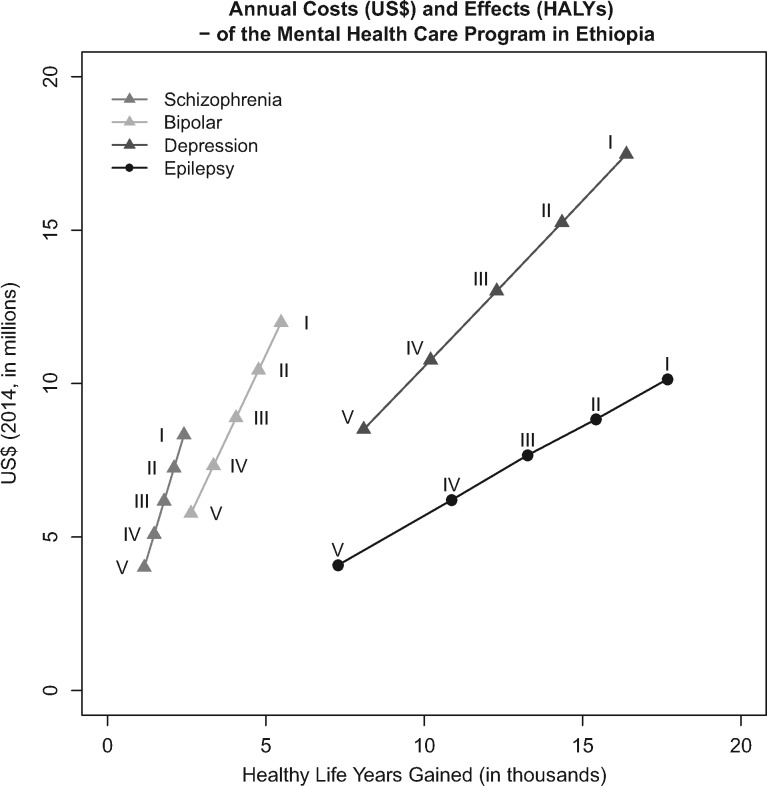
Level and distribution of expected healthy life years gained and programme costs (2014 US$) with the introduction of universal public finance of treatment for depression, bipolar disorders, schizophrenia and epilepsy according to the National Mental Health Strategy in Ethiopia (I is the poorest quintile and V the riches quintile).

The results shown in Table 2 and Figure 1 indicate that the health benefits of the MN intervention packages are expected to be progressive. The poorest quintile is expected to gain 41 970 healthy life years in total for all MN treatments, whereas the richest quintile has an expected gain of 19 160 healthy life years in total. The low-income groups gain more healthy life years than the richest quintiles due to the high disease burden in the lower income quintiles. Total cost of care is also higher in the poorest groups due to the relatively high treatment demand in these groups. The total annual cost of MN health care is expected to be close to US$48 million in the poorest quintile and US$22 million in the richest quintile. Similarly, the measured value of insurance is highest among the lowest income group. Per invested US$1 in MN services in Ethiopia, the expected FRP return is not more than US$0.00001.

The return on productivity from investing in MN health care in Ethiopia seems to be substantially higher than the expected FRP. From the results shown in Table 3, we see that scaled-up depression treatment at 30% coverage could lead to total productivity gains of around US$50.7 million per year. The largest benefits accrue to the wealthier quintiles on account of their higher average income level. Our estimates indicate that the expected productivity gain from scaled-up treatment of depression is likely to reduce the governmental cost of the depression treatment programme by close to 78%.

## Discussion

The ECEA methodology is a novel approach to the economic analysis of mental health policies. It offers quantitative insights on how MN interventions impact several important equity outcomes. This analysis finds that health gains and productivity gains seem to be the most important benefits from scaled-up universal public finance of treatment for epilepsy, depression, bipolar affective disorder and schizophrenia in Ethiopia. Public finance of these MN services yields little prevention of impoverishment due to the low current level of private OOP health care spending. The main reason for this is simply that patients are not impoverished in this way to start with since MN services in Ethiopia are not available for most patients. However, the private household economy seems to indirectly benefit substantially from increased household income. Patients with depression are expected to increase their income when offered depression treatment as they will be less absent from work and more productive when they are at work.

Our results show a large expected increase in healthy life years if the goals of the National Mental Health Strategy in Ethiopia is achieved. That is, to substantially expand coverage of essential treatment for schizophrenia (75% target coverage), bipolar disorder (target coverage 50%), depression (30% target coverage) and epilepsy (target coverage 75%). Good health, or health benefits, is an important social good in itself. The WHO Consultative Group on Equity and Universal Health Coverage identified health benefits to be more important than financial risk protection (World Health Organisation 2014). This international group of ethicists considered it ethically unacceptable to give high priority to costly services that are expected to provide large FRP and small health benefits compared to less costly services that provide substantial health benefits and low FRP (World Health Organisation 2014).

The ECEA methodology uses the current utilization of MN services as reference to how much FRP one can expect from a universal public finance of MN treatment. Public finance of MN services does therefore not seem to protect households in Ethiopia from financial risks. Similarly, low levels of FRP from public finance of antiepileptic drugs in India have been found (Megiddo *et al*. 2016). Pneumococcal vaccines have an estimated insurance value of US$66 000 at an 80% coverage according to a recent study from Ethiopia (Johansson *et al*. 2015), substantially higher than the expected total FRP of US$1,720 from the National Mental Health Strategy in Ethiopia. Universal public finance for tuberculosis treatment in India is expected to give an insurance value of US$9,000, where 80% of it would benefit the two poorest quintiles (Verguet *et al*. 2015a). An ECEA from South Africa estimated households to save US$0.29 per capita in OOP expenditures with an investment of US$0.01 in a salt reduction policy (Watkins *et al*. 2016). The high FRP from salt reduction is mainly due to the high current private OOP spending on cardiovascular disease care in South Africa that will be averted from the salt policy. Similar findings are found in an Ethiopian ECEA, where interventions that avert the most cases of poverty are a universal public finance of interventions with a high level of OOP expenditures (caesarean section, tuberculosis treatment and antihypertensives) (Verguet *et al*. 2015b).

However, scale-up of MN care in Ethiopia is estimated to give a high return in productivity gains. A recent global return on investment analysis estimated an economic return of US$2.3-3.0 in productivity gain per US$1 invested in treatment of depression and anxiety in 36 countries between 2016 and 2030 (Chisholm *et al*. 2016). This global estimate is higher than our expected overall return rate of around US$0.8 per US$1 invested in depression treatment in Ethiopia. We apply modest assumptions on disability days averted to not overestimate the economic return. There is limited evidence from low-income settings on days out of role in low- and middle-income countries. We use average estimates that are derived from contexts that are very different from the Ethiopian and there may be major differences in occupational profile, among other. Depression treatment programmes in United Kingdom (Clark *et al*. 2009) and the United States (Goetzel *et al*. 2003) are estimated to offer somewhat higher productivity gains than our estimates from Ethiopia. More work is needed for developing new methods to estimate the productivity impact of MN interventions in low-income settings where the context is vastly different to high-income settings. Methods need to be sensitive to heterogeneous populations where the majority live in remote rural settings and the rich middle class live modern urban lives. More empirical evidence on how MN disorders de-facto influences productivity in these settings is needed.

ECEA seems to be a feasible approach and a useful addition to policy decision-making, particularly since it builds on existing cost-effectiveness modeling frameworks. In our study, we used the planned coverage rates presented in the National Mental Health Strategy in Ethiopia and scaled them up over a 10-year period. The ministry of health in Ethiopia has an ambitious strategy for mental health care in the country, and we do not evaluate the feasibility of their goals. It will be especially hard to reach the high target coverages in rural parts of Ethiopia, where the majority of the population live, and it will probably take >10 years to reach full coverage of all services. Nonetheless, our results indicate that the expected health and productivity gains are important returns to strive for.

This ECEA is subject to the inherent uncertainty surrounding population-level projections of intervention costs, impacts and consequences, consideration of which is contained in the primary analyses underlying the base case. Therefore, our findings from the application of ECEA to the original CEA of MN disorders need to be interpreted with a due degree of caution. The uncertainty of the results in the existing CEA was in particular noteworthy for interventions targeting schizophrenia and bipolar disorder due to lack of evidence (Strand *et al*. 2015). A method for systematically handling uncertainty in ECEAs is not developed yet. In general, presentation and interpretation of a sensitivity analysis for stratified analysis of multiple parameters and outcomes are complex.

Variations of outcomes in this analysis are driven by the variation of disease prevalence (Fekadu *et al*. 2014) and income level for five income groups (World Bank and World Development Indicators). A socioeconomic gradient is likely to exist for other parameter inputs as well, but such stratified evidence is limited and we therefore decided to hold values of other input parameters fixed across income groups. For MN health care one could expect that it is costlier to reach the poor than the rich with care, the current and target coverage is higher among rich than poor, and the total OOP spending on MN health care and efficacy of interventions will vary across income groups. The stratified results for healthy life years gained, FRP, total governmental costs and private expenditures averted are sensitive to variations in all these parameters: A higher cost to reach the poor with services would increase the total governmental cost, a higher increase in expansion of coverage for the rich would reduce expected health benefits for the poor and increase expected health benefits for the rich, and a higher total OOP expenditure would increase the FRP of the National Mental Health Strategy in Ethiopia. To be able to quantify the impact of these variations, better evidence on the distribution of such policy-relevant parameters and more systematic methods for doing sensitivity analysis in ECEA are needed. The main data requirements for conducting more precise ECEAs are stratified epidemiological and economic input parameters. Such information may be available in national demographic and health surveys, or could be built into future data collections.

## Conclusion

Findings from this ECEA indicate that investing in universal public finance of public mental health will create substantial health benefits and high productivity gains, but it will most likely produce a low degree of FRP. Accordingly, while the ECEA approach captures FRP and equity in the economic evaluation of mental health policy, the FRP benefits are less relevant when the current utilization and spending on care is extremely low, as they are in Ethiopia. Nevertheless, we expect that many families experience impoverishing loss of income because of mental disorders.
